# Intragroup Judgements of Norm Violators Under the Threat of COVID-19

**DOI:** 10.3390/bs16081382

**Published:** 2026-08-11

**Authors:** Arabella Sinclair-Thompson, John A. Hunter

**Affiliations:** Department of Psychology, University of Otago, Dunedin 9054, New Zealand; sinar280@student.otago.ac.nz

**Keywords:** intragroup processes, pandemic threat, norm violation, Black Sheep Effect

## Abstract

The Black Sheep Effect (BSE) posits that deviant in-group members are judged more harshly than deviant out-group members. The present investigation sought to delineate the relative contribution of both group identity and norms to the expression of the BSE. Two studies were conducted in the context of threats (i.e., pathogen intensity and variations in group norms) induced during the COVID-19 pandemic. Each study tested three basic hypotheses. First, that norm violators (i.e., black sheep) would be more negatively evaluated than norm followers. Second, that both group identity and norms would be associated with the negative evaluations of norm violators. Third, that norm violators would be judged more harshly by those who experience the greatest threat (i.e., via pathogen intensity in Study 1 and norm strength in Study 2). Both studies revealed that in-group members who violated COVID-19 norms were judged more harshly than in-group members who adhered to these norms. No evidence was found to show that social identity or threat (as variously manipulated) impacted the BSE. Norms, however, did make a modest contribution to explaining the BSE in both studies. Study 2 revealed that only prescriptive norms were associated with the expression of the BSE. These findings were confirmed in re-analysis of the results found in Study 1. These findings support the idea that prescriptive rather descriptive norms drive the BSE.

## 1. Introduction

The tendency for people to favour in-group members over out-group members is ubiquitous (Brewer, 2017; Brown & Pehrson, 2020; Tajfel et al., 1971). However, although it has received less attention in the literature, there is precedence for category members to sometimes manifest more negative evaluations of in-group members than out-group members (Branscombe et al., 1993; Marques et al., 1988). Responses of this nature are subsumed under the Black Sheep Effect (BSE, Marques et al., 1988). Research shows that the BSE emerges when in-group members act in ways that are judged to be unlikeable, immoral, or violate the social norms of the in-group (Marques & Pinto, 2023). Further, these effects are robust in so far as it has been demonstrated amongst adults and children, people from different countries, and with respect to diverse social behaviours (Iyer et al., 2012; Marques et al., 1988; Pinto et al., 2010; Wu et al., 2019; Yudkin et al., 2020).

A variety of accounts have been offered to explain the BSE (see Tang et al., 2023). One of the most prominent though has emerged from the social identity perspective (an amalgamation of social identity (SIT; Tajfel & Turner, 1979;) and self-categorization theories (SCT; Turner et al., 1987)). According to this framework, the BSE is a function of two broad psychological processes. The first draws on the premise that people are motivated to establish a positive social identity. The assumption is that the BSE is essentially a “sophisticated” form of in-group favouritism (Marques et al., 1988, p. 5), whereby the denigration of “deviant” in-group members serves to maintain a positive view of the in-group overall. The second draws on the assumption that category members are motivated to maintain the integrity of the in-group’s norms (the rules and standards that guide the groups actions). In-group norms are crucial to the behaviour of social category members. In-group members who undermine group norms threaten the cohesiveness of the in-group and ultimately the group’s goals. Thus, the BSE reflects a strategy by which category members seek to defend the validity of the in-group’s norms (Marques & Pinto, 2023).

Research is generally consistent with the SIT approach in so far as experimental research shows that both group identity and social norms impact on the expression of the BSE (Abrams et al., 2013; Castano et al., 2002; Marques et al., 1998; Marques et al., 2001). Whilst such findings have undoubtedly contributed to our understanding of the mechanisms that drive the BSE, a problem with this work is that almost all of it tends to focus on either group identity or norms (Marques et al., 1998). As both are assumed to contribute to the BSE, the emphasis on one as opposed to the other obscures a comprehensive understanding of this phenomenon. This is especially so in light of theory and research, which suggest that norms and group identity overlap (Postmes et al., 2005). Norms define what a group identity means (Morton, 2011), and adhering to group norms is a way in which group members express their social identity (Packer et al., 2021). Moreover, recent work shows that norms and group identity “reciprocally influence” one another over time (Rathbone et al., 2023). Thus, there is reason to believe that the respective contributions of norms and group identity to the BSE may be conflated. As such, one of the primary aims of the present investigation is to delineate the relative contribution of both group identity and norms to the expression of the BSE.

A second aim of this investigation is based on the social identity threat literature (Ellemers et al., 1999). This research typically distinguishes between threats that emerge from the out-group (intergroup threats) and threats that emerge from the in-group (intragroup threats). The evidence suggests that both impact the BSE (Marques et al., 1988; Marques & Pinto, 2023). In the current investigation, we seek to extend this literature by assessing the intragroup threat in the broader context of the COVID-19 pandemic. The threat of death and disruption posed by COVID-19 constitutes what Jutzi et al. (2023) refer to as an existential threat (i.e., large scale death). When threats of this nature are encountered, group- and social identity-based processes typically become paramount (e.g., Jetten et al., 2020; Packer et al., 2021; Van Bavel et al., 2020). Indeed, the data indicate that, in such circumstances, people are more likely to (a) identify with social groups (Hogg, 2009) and (b) adhere to the group’s respective norms (Fritsche et al., 2011; Stollberg et al., 2017; Van Bavel et al., 2022). Thus, in the context of the COVID-19 pandemic we might expect that group identification and adherence to norms are especially likely to be associated with the BSE.

Leveraging the pathogen threat present in the COVID-19 pandemic appears to present a unique opportunity to investigate the mechanisms behind the BSE in circumstances that have rarely been explored in social psychological research. Whilst the present study seeks to do just this, it is important to recognize, as Jutzi et al. (2023) note, that COVID-19 not only fosters existential dread but induces a variety of additional threats that provoke uncertainty (e.g., unemployment, loneliness, the emergence of more dangerous mutations of COVID-19, the disruption of group norms). Consequently, a final aim of the current study is to explore the ramifications of two such threats—pathogen intensity and strength of in-group norms. To this end, two studies were conducted. The first examined the relative contribution of norms and group identity to the BSE as a function of COVID-19 intensity. The second examined the relative of contribution of norms and group identity to the BSE as a function of norm intensity.

## 2. Study 1

### 2.1. Research Question and Hypothesis

In study one, we test three hypotheses. First, we predict that norm violators (i.e., black sheep) will be more negatively evaluated than norm followers (H1). Second, we predict that both group identity and norms will be associated with the negative evaluations of norm violators (H2). Third, we predict that norm violators will be judged more harshly by those who experience the greatest threat from COVID-19 (H3).

### 2.2. Materials and Methods

#### 2.2.1. Participants and Design

Participants (*n* = 258) were drawn from a sample of psychology undergraduate students who completed the study online. All received course credit for taking part. One hundred and eighty-nine identified as female and 66 identified as male, and 3 identified as non-binary or chose not to provide information as regards gender identity.

In a mixed-model design, participants were randomly assigned to one of three conditions (COVID-19 threat: High vs. Low vs. Baseline) before evaluating targets that either adhered to or violated COVID-19 safety norms. Each cell comprised 84–86 participants. Sensitivity analysis conducted using G*Power version 4 (Faul et al., 2009), with the alpha set at 0.05 and power at 0.95, suggests that our sample was of sufficient size to detect a small effect (Cohen’s *d* = 0.25).

#### 2.2.2. Procedure

The study was introduced as being concerned with the attitudes of New Zealanders during the COVID-19 pandemic. After collecting demographic data on ethnicity, gender identity, and secondary school decile, participants were presented with the 14-item in-group identification scale (e.g., “Being a member of this group is an important part of how I see myself”, Cronbach’s alpha = 0.92, Leach et al., 2008) and were asked to respond in terms of their membership in the New Zealand national group. COVID-19 norms were assessed using a 4-item “community norms” measure adapted from Dhar et al. (2018). Two of the items focused on prescriptive norms, (i.e., “I believe COVID-19 safety guidelines should be followed”, “I think it would be okay to criticize someone for ignoring COVID-19 safety guidelines”) and two focused on descriptive norms (i.e., “I believe that most people in New Zealand follow COVID-19 safety guidelines”, “I believe my community would think poorly of someone who ignored COVID-19 safety guidelines”, Cronbach’s alpha = 0.64).

In order to ensure that participants’ evaluations of targets were not attributable to other constructs that have been found to be related to judgements of group members and reactions to the COVID-19 pandemic, we included the following scales. Fear of COVID-19 was measured via two items (i.e., “It makes me uncomfortable to think about Coronavirus-19”, “When watching news and stories about Coronavirus-19 on social media, I become nervous or anxious”, Cronbach’s alpha = 0.82, Winter et al., 2023). Social disgust was measured on the 13-item Disgust scale (e.g., “If I see someone vomit, it makes me sick to my stomach”, Cronbach’s alpha = 0.76, Olatunji et al., 2007). Right-wing Authoritarianism was assessed using the “Authoritarianism, Conservatism, and Traditionalism” (ACT) scale, consisting of 36 items (e.g., “Capital punishment is barbaric and never justified”, Cronbach’s alpha = 0.91, Duckitt et al., 2010). Social Dominance Orientation was assessed using the SDO_7_ scale consisting of eight items (e.g., “It is unjust to try and make groups equal”, Cronbach’s alpha = 0.87, Ho et al., 2015). Responses to all scales were scored on a seven-point Likert scale (1—strongly disagree; 7—strongly agree) and in terms of how participants felt at the time of the study.

Participants were then presented with one of three vignettes. Participants in the High threat condition were, using a widely utilized method (Bain et al., 2013; Duckitt & Fisher, 2003), asked to imagine life in New Zealand during a hypothetical outbreak of a new, more dangerous variant of COVID-19. In this instance, a higher mortality and infection rate was emphasised, along with more severe symptoms. Participants in the Low threat condition were asked to imagine life in New Zealand during a hypothetical outbreak of a less deadly variant of COVID-19. In this condition, a mortality and infection rate half that of the original virus was emphasised, as well as much milder symptoms. Finally, participants in the Baseline condition were presented with a vignette asking them to imagine the next year of the pandemic continuing in New Zealand in much the same way it has thus far. This primer described the possibility that new variants may occasionally enter the country, but that generally they will be prevented from entering communities through self-isolation and well-practiced contact tracing protocols. After reading the vignette, participants were presented with five manipulation check questions. In order to assess whether the different conditions effectively elicited different levels of threat, participants rated the imagined scenario they were presented with in terms of how threatening, hard, frightening, competitive, and uncertain they believed them to be. Each aspect of the manipulation check was scored on a nine-point Likert scale (1—strongly disagree; 9—strongly agree).

Following the manipulation, participants in each condition (High threat, Low threat, and Baseline) read several, randomly presented pairs of vignettes describing different in-group target’s behaviour during various pandemic situations (i.e., working while sick, attending a party with no social distancing, running an errand, and attending a date while sick). One article in each pair describes a norm adhering (NA) target (e.g., John from Tauranga) that finds itself in a difficult situation during the pandemic (e.g., becoming sick before an important work meeting) and emphasizes the author’s adherence to the national COVID-19 protocol (e.g., staying home while sick, social distancing, the use of contact tracing). The other article describes a norm violating (NV) target (e.g., James from Taupo) that finds itself in a similar pandemic conundrum but emphasizes the target’s choice to ignore national COVID-19 protocol (e.g., goes to work while sick, ignores social distancing, refuses to use contact tracing). In total, participants read eight vignettes (four male targets and four female targets) encompassing four events. Two events involved female targets and two involved male targets. All the names used to depict the vignette targets were sourced from Hunter et al. (1997), who showed that they were perceived as equally positive.

After reading each article, participants rated each target using 20 pairs of 9-point trait-ratings scales. The terms (e.g., ignorant–well-informed, honest–dishonest, selfish–unselfish, straight forward–hypocritical) used in these scales were adapted from Hunter et al. (2017). Responses were coded so that high scores reflected positive evaluations.

## 3. Results

### 3.1. Preliminary Analysis and Manipulation Checks

Following the various recommendations outlined by Field (2013), all data initially were checked for normality, homogeneity of variance, and outliers. Preliminary analysis revealed a marginal effect for gender identity with respect to judgements of norm violators. Because there was not sufficient statistical power to draw any valid conclusions from analyses using the three participants (who respectively identified as non-conforming, non-binary, and preferred not to give any information as regards gender), we opted to remove these data points. Upon further analysis, the removal of these participants (two of whom gave especially high evaluations of NA’s *M* = 8.24 and low evaluations of NV’s *M* = 3.74; the other evaluated NV’s more positively than NA’s, *M* = 5.73 vs. *M* = 5.64) completely attenuated the gender effect. This finding supports the view that the inclusion of small sample sizes and the *p* values that go along with them are often unreliable (Cao et al., 2024). We subsequently did not include gender in the following analyses. We acknowledge the limitations of our data as regards inclusivity and support, and with all attempts, we gather larger samples that comprise such individuals in the future.

A between-groups MANOVA was conducted to assess the success of the threat manipulation. Cell means may be seen in Table 1. A main effect was found for condition, *F*(10, 496) = 47.72, *p* < 0.001; Wilks’ Lambda = 0.258, *η*^2^ = 0.491. Univariate ANOVA’s revealed significant effects for life being perceived as threatening, *F*(2, 251) = 259 0.12, *p* < 0.001, *η*^2^ = 0.67, hard, *F*(2, 251) = 198.55, *p* < 0.001, *η*^2^ = 0.61, frightening, *F*(2, 251) = 204.85, *p* < 0.001, *η*^2^ = 0.62, competitive, *F*(2, 251) = 99.81, *p* < 0.001, *η*^2^ = 0.44, and uncertain, *F*(2, 251) = 120.94, *p* < 0.001, *η*^2^ = 0.49. Planned comparisons (incorporating the Bonferroni correction) revealed that in the High-threat condition, life was perceived to be more threatening, harder, frightening, competitive, and uncertain than in both the Low threat and Baseline conditions (all *p*’s < 0.001). The Baseline condition was also perceived as more threatening than the Low-threat condition across all manipulation checks (all *p*’s < 0.001).

#### 3.1.1. Main Analysis

Preliminary analyses revealed that norm adherents (NA) and norm violators (NV) were judged in an identical fashion across each of the four events (each NA was rated more positively that each NV, all *p*’s < 0.001). Thus, for simplicity, and following the standard procedure in this field (see Islam & Hewstone, 1993; Hunter et al., 2000; Hunter et al., 2026), we combined responses across events into one overall score for NA and one overall score for NV. To investigate the effect of COVID-19 threat conditions (High, Low, or Baseline) on participants’ evaluations of NA and NV targets, we conducted a 3 (COVID-19 threat: High, Low, Baseline) × 2 (Target: adherent vs. violator) mixed-model ANOVA. Cell means may be seen in Table 2. The first factor was between subjects. The second was within subjects. The only effect to emerge was a main effect for Norms, *F*(1, 252) = 704.38, *p* < 0.001, *η*^2^ = 0.737. NA targets were evaluated much more positively than NV targets (M = 6.70, SD = 0.89 vs. M = 4.37, SD = 0.78).

#### 3.1.2. Associations Between the Variables

In an attempt to examine the extent to which New Zealand national social identity, norms, fear of COVID-19, disgust, RWA, and SDO contributed to evaluations of NA and NV targets, we constructed an index of target evaluations. This was achieved by subtracting evaluations of NV targets from evaluations of NA targets. Correlations between the index of evaluations and each of the other variables may be seen in Table 3.

As may be seen from Table 3, many of the variables are related. Target evaluations are negatively associated with SDO and positively related to fear of COVID-19, disgust, social identity, and norms. To tease apart the relative contribution of each of the variables, a standard multiple regression was conducted. As may be seen in Table 4, the overall regression was significant, *R*^2^ = 0.12, *F*(6, 248) = 5.49, *p* < 0.001. Moreover, inspection of beta weights revealed that only acceptance of norms made a unique contribution to the evaluative index.

Following the recommendations of several researchers (Nathans et al., 2012; Pedhazur, 1997; Tabachnick & Fidell, 2007, pp. 144–145) who raise concerns pertaining to the over reliance on beta weights in regression analyses, we additionally assessed the association between evaluative judgements and norms via partial correlation. This analysis confirmed that the link between norms and evaluative judgements remained significant when all other variables were controlled.

### 3.2. Conclusion Study 1

Support was found for the first hypothesis, as NA targets were consistently judged more positively than NV targets across all four vignette events. Limited support was found for the second hypothesis, and no support was found for the third hypothesis. Regarding the second hypothesis, there was no significant association found between in-group identification and judgements of vignette targets. A relationship was, however, found between judgements of NV targets and norms. While correlational analysis initially revealed a positive correlation between both social identity and norms and the differential evaluation of NA and NV targets, this correlation only held for norms significant when each of the covariates was included. With respect to the third hypothesis, although manipulation checks revealed that participants in the High-threat condition felt significantly more threatened than those in the Low-threat or Baseline conditions, participants across all three conditions evaluated NV targets more negatively than NA targets. Thus, in spite of our successful threat manipulation, perceptions of higher threat were not significantly associated with harsher judgements of NV targets.

The limited support found for our second and third hypotheses is at odds with research suggesting that, during periods of threat, highly identified in-group members will make use of the BSE. Branscombe et al. (1993) found that only highly identified participants expressed extreme negative opinions about other in-group members, and that these negative evaluations were exacerbated under conditions of high threat.

Our analyses nevertheless revealed that participants’ endorsement of COVID-19 norms was significantly linked to the differential evaluation of NV and NA targets. These findings are consistent with recent research suggesting that norms become highly salient during periods of threat and may be an especially important predictor of the BSE (Marques & Pinto, 2023; Van Bavel et al., 2022). Indeed, the results of the present study suggest that the awareness and acceptance of the social norms is a more significant predictor of evaluations of this effect than in-group identification, and other variables such as RWA, SDO, fear of COVID-19, and social disgust.

Although the lack of effects for social identity was not anticipated (but not precedented (see Hinkle & Brown, 1990; Turner, 1999; Hunter et al., 2026)), our results underline the importance of norms. Whilst this latter outcome is consistent with much previous research and theory (Marques et al., 1998; Marques & Pinto, 2023), the current study is the first to compare the relative contribution of both social identity and norms. Considering the significance of norms in determining our participants’ reactions to targets’ behaviour during the pandemic, we conducted a second study to further explore the effect of social norms by manipulating participants’ perception of the strength of national norms concerning COVID-19 safety protocols.

Furthermore, although most researchers highlight the importance of norms with respect to in-group behaviour and the BSE, there is disagreement as to the nature of the norms that drive such behaviour. Indeed, with respect to those who work within the SIT framework, some argue that reactions to other in-group members is largely governed by descriptive norms (Rathbone et al., 2023). Others who have advanced this perspective to develop the subjective group dynamics approach (SGD; Marques et al., 1998) argue that prescriptive norms drive the BSE. Thus, in light of these disagreements, we decided to include measures of both descriptive and prescriptive norms in our second study.

## 4. Study 2

### 4.1. Research Question and Hypothesis

The results of Study 1 appear to suggest that acceptance and awareness of social norms have a more powerful influence on the BSE than does social identity or any of the other included covariates. According to Bicchieri (2016) and other scholars (Marques et al., 1998; Marques & Pinto, 2023; Rathbone et al., 2023), social norms comprise both prescriptive (i.e., do you personally believe it is important to adhere to the norm) and descriptive beliefs (i.e., do enough of your peers believe in the importance of the norm, to the point that they would be willing to punish behaviour that deviates from it?). In order to better assess the respective contributions of both prescriptive and descriptive norms to judgements of NV in-group members, Study 2 included an expanded measure of social norms. Although we do not make predictions as to which type of norm might be more important, this measure will be better suited to parse out the relative contribution of each type of norm, thereby allowing us to gather robust data on type of norm that influences the expression of the BSE.

Previous research suggests that behaviour that is motivated by social norms may be manipulated via increasing the salience of the norm in question and altering the normative and empirical expectations of participants (Bicchieri & Chavez, 2010; Jetten et al., 1996). It is predicted that manipulating participants’ empirical and normative expectations will affect their judgement of NV vignette targets. That is, implying wider social acceptance of COVID-19 safety norms will lead participants to judge NV targets more severely. Alternatively, indicating relatively low social acceptance of these norms will lead judgements of violators to be less extreme.

Research Question and Hypothesis:

As in Study 1, we test three hypotheses. First, we predict that norm violators (i.e., black sheep) will be more negatively evaluated than norm followers (H1). Second, we predict that both group identity and norms will be associated with the negative evaluations of norm violators (H2). Third, we predict that norm violators will be judged more harshly in circumstances that mandate strong compliance with COVID-19 safety norms.

### 4.2. Participants and Design

Participants (*n* = 196) were drawn from a sample of psychology undergraduate students (157 identified as women, 37 identified as men and 7 as non-binary). All received course credit for participation.

Using a mixed-model design, participants were randomly assigned to one of three conditions (Norm manipulation: Strong norm vs. Weak norm vs. Control) before reading and responding to several vignettes depicting targets who either adhered to or violated COVID-19 safety norms. Each cell comprises around 66 participants. Sensitivity analysis conducted using G*Power (Faul et al., 2009), with the alpha set at 0.05 and power at 0.95, suggests that our sample was of sufficient size to detect a small effect (Cohen’s *d* = 0.41).

### 4.3. Procedure

The study was introduced as being concerned with the impact of personality and identity on social judgements in Aotearoa New Zealand. Demographic data on ethnicity, gender identity, and secondary school decile were collected before participants were presented with the same scales used in Study 1. Participants were then presented with one of three manipulations. Those assigned to either the Strong or Weak norm conditions were presented with a brief paragraph describing a bogus study investigating the number of people in Aotearoa New Zealand who supported and complied with COVID-19 safety mandates that was said to have been conducted in 2021. In the Strong norm condition, the study was reported to have found that 9 out of every 10 New Zealanders fully supported the mandates and followed them to the letter every day. While in the Weak norm condition, it was reported that only 2 out of every 10 New Zealanders completely supported the mandates and adhered to them every day. After reading these fabricated reports, participants completed a manipulation check that asked whether they got the impression that the majority of New Zealanders follow COVID-19 safety mandates every day. The manipulation check was scored on a seven-point Likert scale (1—strongly disagree; 7—strongly agree). Those assigned to the Control condition were shown a screen thanking them for completing the study thus far and asking them to continue with the next phase of the survey, receiving no manipulation.

After the manipulation, participants read several, randomly presented pairs of vignettes describing the behaviour of eight in-group targets across four situations occurring during the COVID-19 pandemic. These vignettes were identical to those presented in Study 1, save for one new vignette (i.e., choosing whether or not to isolate while a housemate waits for a test result) that replaced the “errand run” vignette from the first study. This vignette was removed as it mainly grappled with the choice of whether or not to wear a mask on public transit or in a supermarket, and over the course of the pandemic, mask wearing in shops and on busses had become mandatory, essentially making the original vignette obsolete.

Following each vignette, participants rated each target using the same 20 pairs of 9-point trait rating scales utilised in study one. Responses were coded so that high scores represented more positive evaluations of targets. Also included was a six-item expanded norms measure, adapted from Bicchieri (2016). This particular scale sought to assess both descriptive and prescriptive norms. Three items assessed awareness of and adherence to descriptive COVID-19 safety norms (e.g., “I believe that most people in New Zealand support the importance of COVID-19 safety guidelines”, Cronbach’s alpha = 0.82). The remaining three items comprised assessed awareness and adherence of prescriptive norms (e.g., “I think it would be okay to criticize someone for ignoring COVID-19 safety guidelines” Cronbach’s alpha = 0.71). All items were scored on a seven-point Likert scale (1—agree strongly; 7—disagree strongly), and with respect to feelings at the time in questions. 

## 5. Results

### 5.1. Manipulation Checks

The difference between responses to the Strong and Weak norm manipulation check questions was significant at *p* = 0.021. Participants in the Strong norm condition reported a significantly higher agreement with a statement suggesting that the majority of New Zealanders completely support the COVID-19 safety protocol, as opposed to those in the Weak norm condition (M = 5.15, SD = 1.56 vs. M = 4.48, SD = 1.70, *F*(1, 126) = 5.47, *p* = 0.021, *η*^2^ = 0.041).

### 5.2. Main Analysis

As in Study 1, and following the recommendations outlined by Field (2013), preliminary analyses revealed that norm-violating targets were judged more negatively across all four events (all *p*’s < 0.001). Furthermore, no main or interaction effects were found with respect to gender identity. In the absence of any meaningful effects, and in order to simplify analyses, responses were combined across vignette events to create an overall “norm-adherent” (NA) and “norm-violating” (NV) judgement score. Utilizing these judgement scores, a 3 (Norm manipulation: Strong, Weak, Control) × 2 (Target: adherent vs. violator) mixed-model ANOVA was conducted to evaluate the effect of the norm manipulation on responses to NA and NV vignette targets. Cell means may be seen in Table 5.

The first factor was between subjects, and the second was within subjects. The only effect that emerged was a main effect for target, *F*(1, 193) = 668.87, *p* < 0.001, *η*^2^ = 0.776. Norm adherent targets were evaluated much more favourably than norm-violating targets (M = 6.94, SD = 0.92 vs. M = 4.36, SD = 0.72).

As in Study 1, an index of target valuations was constructed by subtracting the overall NV evaluation score from the overall NA score. This index of evaluations was then utilized in a correlational analysis to determine the relationship between target evaluations, social identity, fear of COVID-19, disgust, RWA, SDO, and both the personal and social norm-acceptance subscales.

Correlations between the index of target evaluations and each of the variables described above may be seen in Table 6. As may be seen, several of the variables are significantly related. The prescriptive norm subscale in particular is significantly positively associated with target evaluations. Several variables associated with target evaluations came close to reaching significance, specifically disgust, social identity, and SDO. In order to further assess the relationships between target evaluations and the variables described above, a standard multiple regression was conducted (see Table 7). The index of target evaluation was entered as the dependent variable, while RWA, disgust, fear of COVID-19, social identity, SDO, and both of the prescriptive and descriptive subscales were entered as independent variables. The overall regression was significant *R*^2^ = 0.12, *F*(7, 188) = 3.524, *p* = 0.001. Inspection of beta weights revealed that prescriptive norms and fear of COVID-19 were the only significant predictors of target evaluation. Further analysis using partial correlations confirmed that these associations remained significant when all other variables were controlled.

### 5.3. Discussion Study 2

As in Study 1, NA targets were evaluated significantly more positively than NV targets across all vignette events. Our manipulation checks revealed that our norm manipulation was successful as participants in the Strong norm condition reported significantly higher agreement with a statement claiming that the majority of New Zealanders follow COVID-19 protocol every day than those in the Weak norm condition. However, it was not successful at affecting participants’ judgements of NV targets, as no significant difference in judgement was found between those in the Strong norm, Weak norm, or Control conditions. Therefore, despite our successful manipulation, no support was found for our hypothesis.

Although our norm manipulation did not impact participants’ target evaluations, norm awareness continued to be the most consistently significant predictor of participants’ evaluations. As in Study 1, our norm measure was significantly related to several variables. In fact, Study 2’s expanded-norms measure revealed that the prescriptive norm subscale was significantly linked to the differential evaluation of NV and NA targets. This suggests that participants’ endorsement of a strong personal commitment to following COVID-19 safety protocol, and a personal willingness to sanction behaviour that violates that protocol (i.e., prescriptive norms), was a stronger predictor of their judgement of NV targets than identification with the New Zealand in-group, RWA, SDO, or even their awareness of the number of other New Zealanders who supported COVID-19 safety guidelines (i.e., descriptive norms).

Fear of COVID-19 also appeared to be significantly negatively linked to target evaluations in the Study 2 multiple regression analysis. However, this link needs to be treated with caution as it did not appear to be significant in Study 2’s correlational analysis, or in Study 1’s regression analysis. The consistency and strength of the association between norm awareness and target evaluation across both studies suggests that it is a more meaningful predictor of the differential evaluation of NV and NA targets.

## 6. Discussion

The present investigation sought to delineate the relative contribution of both group identity and norms to the expression of the BSE in the context of the threats (i.e., pathogen intensity and variations in group norms) induced during the COVID-19 pandemic. Two studies were conducted. Each tested three basic hypotheses. First, that norm violators (i.e., black sheep) would be more negatively evaluated than norm followers. Second, that both group identity and norms would be associated with the negative evaluations of norm violators. Third, that norm violators would be judged more harshly by those who experience the greatest threat (i.e., via pathogen intensity and norm strength).

Both studies revealed that in-group members who violated COVID-19 norms were judged more harshly than in-group members who adhered to these norms. This indicates clear evidence for the BSE. No evidence was found to show that social identity or threat (as variously manipulated) impacted on the BSE. Social norms, however, did make a modest contribution to explaining the BSE in both studies. Study 2 revealed that only prescriptive norms were associated with the expression of the BSE. Such findings prompted a re-analysis of the results of Study 1. This (post hoc) analysis was conducted by separating the four-item norm measure used there into two sub-scales. One focused on two items targeting prescriptive norms (i.e., “I believe COVID-19 safety guidelines should be followed”, Cronbach’s alpha = 0.74). The other focused on two items targeting descriptive norms (“I believe that most people in New Zealand follow the COVID-19 safety guidelines”, Cronbach’s alpha = 0.58). Another multiple regression analysis was subsequently conducted. The results, which may be seen in Table 8, suggest that prescriptive rather than descriptive norms were associated with the BSE. Thus, these findings provide further support for the effects reported in Study 2.

Although our conclusions need to be tempered by the less than adequate reliability of the descriptive norm (α = 58) sub-scale used in Study 1, the fact that prescriptive sub-scales were relatively reliable (Study 1, α = 74; Study 2, α = 71), and that prescriptive as opposed to descriptive norms were related to the BSE, is consistent with the theoretical arguments of Marques et al. (1998). These researchers, via the SGD, argue that prescriptive norms are especially relevant to the BSE. Descriptive norms, they suggest, merely help to differentiate the in-group from the out-group. Prescriptive norms, on the other hand, are vital because they validate the subjective reality of the in-group. Consequently, those who violate these norms threaten the validity of group norms (together with its cohesion and goals), and so they are treated harshly (Marques & Pinto, 2023).

If the assertions behind Marques and colleagues’ (Marques et al., 1998) SGD model are correct, this would help explain why the descriptive norm manipulation utilised in Study 2 failed to impact the expression of the BSE. Previous research suggests that if behaviour is motivated by descriptive norms, changes to these expectations should affect participants’ willingness to adhere to the norm in question (Bicchieri & Chavez, 2010). However, both SGD (Marques et al., 1998) and Bicchieri (2016) propose that if a specific norm is prescriptive, then it will be the most important predictor as regards to whether individual behaviour will align with or deviate from a given norm.

As New Zealand faced higher levels of threat during the global pandemic, the importance of adhering to social norms that encourage cooperation with pandemic safety regulations may have increased, resulting in the significant association between our prescriptive norm scales and judgements of NV targets. It is possible that as COVID-19 continues to pose significant risks to health and wellbeing, the prescriptive social norms encouraging cooperation with national public health guidelines may have become so salient that people have begun to incorporate them into their personal value systems, potentially explaining why these norms are so resistant to manipulation.

These findings have important implications for the motivations behind whether or not an individual chooses to comply with public health guidelines and may be beneficial for future work aiming to increase engagement with society-wide initiatives that require the cooperation of everyone involved. A possible explanation for the integration of norms involving COVID-19 safety guidelines into participants’ prescriptive beliefs may be found in Gelfand et al.’s (2011) discussion of social threat and cultural understandings of the value of norm following. Gelfand describe a spectrum of cultural “tightness” and “looseness”, wherein “tight” cultures value in-group cohesion and readily punish norm violation. Loose cultures, on the other hand, prioritise individual self-expression and are less likely to punish deviation from traditional norms. Gelfand et al. (2011) found that in cultures where there was a history of ecological and man-made threats (e.g., natural disasters, territorial conflict), strong social norms and in-group cohesion tended to develop. The rationale is that these factors would function to facilitate the society-wide co-operation needed to combat these threats.

In light of evidence suggesting that the tightness of prescriptive norms change as a function of context (Szekely et al., 2021), it may be that because New Zealand faced especially high levels of threat during the COVID-19 pandemic, the importance of adhering to norms that encourage cooperation with pandemic safety regulations would likely have increased. This may have resulted in the significant association between our prescriptive norm scales and judgements of NV targets. Additionally, it is possible that as COVID-19 continues to pose significant risks to health and wellbeing, the prescriptive social norms encouraging cooperation with national public health guidelines may have become so salient that people have begun to incorporate them into their personal value systems, potentially explaining why these norms are so resistant to manipulation.

### 6.1. Theoretical Implications

Research on the BSE suggests that level of in-group identification and norms are crucial in determining the severity of one’s judgements of norm violating in-group members (Branscombe et al., 1993; Marques & Pinto, 2023). The findings from both studies presented here found little evidence to implicate in-group identification (when other variables were held constant). From the perspective of SIT, such findings are not without precedent (see Hinkle & Brown, 1990; Hunter et al., 2026; Turner, 1999). However, most BSE studies include a salient out-group to compare evaluations of both in- and out-group targets. It is possible that when the threat operationalized in the study cannot be attributed to a specific out-group, participants are forced to only consider the behaviour of in-group members. In these cases, changes may occur to in-group functioning that affect the factors that significantly impact participants’ evaluations of others. Without a salient out-group, evaluations of NV in-group members may be motivated by prescriptive norms, rather than level of in-group identification or descriptive norms. It is also possible that adherence to prescriptive norms functions to reinforce the group’s social identity. Thus, had we assessed social identity towards the end of the study (i.e., after the expression of the BSE and norm adherence), an effect might have emerged. Nevertheless, it is clear that further work investigating group-based activity in intragroup contexts is needed in order to better understand how group functioning is altered without a comparative out-group.

### 6.2. Limitations

Although our studies present novel findings concerning the motivating factors behind judgements of NV in-group members, our research ran into several limitations during data collection. A consistent limitation with conducting research explicitly tackling COVID-19 safety behaviour in the middle of the pandemic is the fact that the landscape of risk constantly shifts while research is being conducted. Although the High-threat condition from Study 1 elicited significantly higher feelings of threat than the Baseline condition, we noticed that as data collection continued, the experience of COVID-19 in New Zealand began to look more like our imagined High-threat condition, rather than the Baseline condition as originally intended. Because of the shifting levels of risk both nationally and globally, it is possible that our threat conditions were unable to engage participants’ imaginations of a far worse future possibility and instead led them to think about the present state of the country. This may have affected how impactful our threat conditions were at changing participants’ evaluations of targets.

Similarly, the lack of impact from our norm manipulation in Study 2 could have been due to the timing of our research. Study 1 was conducted during lockdown, while Study 2 was conducted post-lockdown, when many New Zealanders were eager to return to “normality” and protests against COVID-19 mandates were beginning to dominate the news. Being unable to anticipate how prevailing attitudes towards COVID-19 safety norms shifted over the course of data collection may have affected how impactful our manipulations were in both studies. The shifting climate during Study 2 may explain the emergence of a slight negative effect of fear of COVID-19 on target evaluations. The Fear of COVID-19 scale (Winter et al., 2023) taps into anxiety around thinking or hearing about COVID-19. It is possible that post-lockdown, participants who prefer not to think about COVID-19 may have been less inclined to negatively evaluate NV targets. Finally, in interpreting the absence of significant effects for our manipulations, we might also note, to paraphrase an anonymous reviewer, they may have been “insufficiently strong or ecologically valid”.

Additionally, while conducting our studies online allowed for data collection to continue over lockdown, it led to several issues that may have impacted the effectiveness of our manipulations. Participants were able to complete the study from their own homes and were able to start and stop the survey as they pleased, with no limits on how long they took to complete it. Unfortunately, this meant some participants may have reached the manipulation stage of the study before pausing the survey and coming back days later to continue and may have forgotten about the primer paragraphs by the time they responded to the vignettes. Furthermore, delivering manipulations in the form of brief paragraphs may have been ineffective due to the nature of online research. Future research aiming to utilize similar manipulations may benefit from delivering it in person, or via a video. In an entirely text-based online survey, we unfortunately had no way of controlling whether participants stopped to take the information on board or simply skimmed over the text before moving on to the next question. Forcing participants to stop and pay attention during the manipulation may greatly improve its efficacy.

In spite of the limitations addressed above, our online study allowed for the collection of data concerning participants’ responses to the COVID-19 pandemic as it was happening. This presented a valuable opportunity to assess the impacts to in-group functioning during a period of unprecedented global threat. Thus, although both our studies ran into setbacks due to the nature of online research and the changing face of the pandemic, the data we were able to collect, and the conclusions we were able to draw, remain interesting and valuable in the face of an ongoing crisis.

## 7. Conclusions

The majority of the research on in-group extremity and the BSE thus far has investigated it in the context of intergroup interactions, comparing participants’ evaluations of both in-group and out-group targets (e.g., Marques et al., 1988; Branscombe et al., 1993). However, as Brewer (2017) suggested, the processes involved in intragroup interaction are complex enough to warrant research that focuses explicitly on the mechanisms at play within a single in-group, as the mere presence of an out-group can induce significant changes to in-group functioning (Tajfel & Turner, 1979; Turner et al., 1987). Therefore, the two studies detailed here, alongside future work in intragroup conditions, have the potential to further our understanding of the complex outcomes of in-group formation and the mechanisms behind conflicts that emerge from within a single group.

Our findings, showing that prescriptive norms appear to be the most significant and consistent predictor of a willingness to sanction the norm violating behaviour of in-group members, have interesting implications for the theories behind intragroup conflict. As these results contrast with much of the research suggesting that high in-group identity is a key factor behind intragroup extremity and the evaluation of disloyal in-group members (Branscombe et al., 1993; Iyer et al., 2012), the findings suggest that future research should investigate whether prescriptive norms continue to predict intragroup judgements across different group types and conditions of threat (see Ellemers et al., 1999; Hinkle & Brown, 1990). Moreover, given the relatively modest contribution of prescriptive norms (i.e., between approximately 8% and 10% of the variance) across both studies, it is important to consider the myriad of other variables that may affect the BSE (e.g., personal values, autonomy, belonging, control).

Recently, Iacoviello and Spears (2022) lamented the dearth of contemporary research into the impact of social norms on both intragroup and intergroup processes. Across several minimal group paradigm studies, they found that prescriptive norms played a significant role in motivating behaviour towards in-group and out-group members and argued for a return to the study of social norms as a powerful factor influencing group behaviour. We reiterate that it is clear that further research into both social norms and intragroup contexts is warranted in order to delineate the extent to which prescriptive and descriptive norms impact group identity and behaviour during periods of threat.

## Figures and Tables

**Table 1:** Descriptive Statistics from the Manipulation Checks for each Condition.

	Condition	Mean	Std. Deviation	N
Threatening	High threat	8.07 **	1.306	84
	Low threat	2.12	1.831	86
	Baseline	3.54 *	2.091	85
	Total	4.55	3.094	255
Hard	High threat	8.30 **	1.117	84
	Low threat	2.64	1.915	86
	Baseline	4.95 *	2.329	85
	Total	5.27	2.973	255
Frightening	High threat	8.18 **	1.263	84
	Low threat	2.40	1.960	86
	Baseline	4.12 *	2.337	85
	Total	4.87	3.082	255
Competitive	High threat	7.36 **	1.781	84
	Low threat	2.90	2.311	86
	Baseline	4.22 *	2.238	85
	Total	4.81	2.825	255
Uncertain	High threat	8.18 **	1.282	84
	Low threat	3.42	2.267	86
	Baseline	6.07 *	2.267	85
	Total	5.87	2.787	255

Note high scores indicate greater perception of threat, hardship, fright, competition, and uncertainty. ** *p* < 0.001 reflects higher scores in the High threat as opposed to the Low threat and Baseline conditions by Bonferroni *t*. * *p* < 0.001 reflects higher scores in the Baseline as opposed to the Low-threat condition by Bonferroni *t*.

**Table 2:** Descriptive Statistics from NA and NV Target Evaluations.

	Condition	Mean	Std. Deviation	N
NA	High threat	6.73	0.91	84
	Low threat	6.70	0.94	86
	Baseline	6.68	0.83	85
	Total	6.70 **	0.89	255
NV	High threat	4.34	0.87	84
	Low threat	4.32	0.74	86
	Baseline	4.44	0.73	85
	Total	4.37	0.78	255

NA = norm adhering, NV = norm violating. ** *p* < 0.001, more positive evaluation of NA than NV targets by ANOVA.

**Table 3:** Correlations between evaluative index, fear of COVID-19, disgust, RWA, SDO, social identity, and norms.

	Index	Cofear	Disgust	RWA	SDO	Socid	Norms
Index	-	0.129 *	0.154 **	−0.055	−0.156 **	0.128 *	0.282 ***
Cofear	-	-	0.448 ***	0.114 *	−0.100	−0.008	0.079
Disgust	-	-	-	0.252 ***	−0.022	0.108 *	0.040
RWA	-	-	-	-	0.497 ***	0.131 *	−0.084
SDO	-	-	-	-	-	0.105 *	−0.236 ***
Socid	-	-	-	-	-	-	0.187 **
Norms	-	-	-	-	-	-	-

Index = index of evaluative difference between Norm adhering and Norm violating targets, Cofear = Fear of COVID, Disgust—Disgust, RWA = Right Wing Authoritarianism, SDO = social dominance orientation, Socid = social identity, and Norms = norms. * *p* < 0.05, ** *p* < 0.01, *** *p* < 0.001.

**Table 4:** Unstandardized B’s, Betas, zero-order, and partial correlations between fear of COVID-19, disgust, RWA, SDO, social identity, norms, and index of target evaluations.

Model		Unstandardized Coefficients		Standardized Coefficients	*t*	Sig.	Correlations	
		B	Std. Error	Beta			Zero-Order	Partial
1	(Constant)	−90.177	69.594		−1.296	0.196		
	Cofear	1.922	2.362	0.055	0.814	0.417	0.129	0.052
	Disgust	1.305	0.754	0.120	1.730	0.085	0.154	0.109
	RWA	−0.197	0.325	−0.044	−0.609	0.543	−0.055	−0.039
	SDO	−1.075	0.978	−0.080	−1.100	0.272	−0.156	−0.070
	Socid	0.829	0.599	0.086	1.384	0.168	0.128	0.088
	Norms	7.973	2.147	0.234	3.713	0.000	0.282	0.229
Dependent Variable: Index of Target Evaluations								

**Table 5:** Descriptive statistics from the vignette-target evaluations.

	Condition	Mean	Std. Deviation	N
NA	Strong	6.94	0.86	67
	Weak	6.86	0.91	61
	Control	7.01	0.97	68
	Total	6.94 **	0.92	196
NV	Strong	4.22	0.75	67
	Weak	4.38	0.70	61
	Control	4.47	0.71	68
	Total	4.36	0.72	196

** *p* < 0.001, more positive evaluation of NA than NV targets by ANOVA.

**Table 6:** Correlations between RWA, prescriptive and descriptive norms, disgust, fear of COVID-19, social identity, SDO, and index of target evaluations.

		Index	RWA	Prescriptive Norms	Descriptive Norms	Disgust	Fear of COVID-19	Social Identity	SDO
Pearson Correlation	Index	-	0.037	0.255 ***	0.055	0.099 #	−0.052	0.103 #	−0.094 #
	RWA	-	-	−0.232 **	−0.016	0.104 #	−0.033	0.109 #	0.504 ***
	Prescriptive norms	-	-	-	0.405 ***	0.075	0.247 ***	0.024	−0.278 ***
	Descriptive norms	-	-	-	-	0.085	0.019	0.129 *	−0.122 *
	Disgust	-	-	-	-	-	0.189 **	0.110 #	−0.079
	Fear of COVID-19	-	-	-	-	-	-	−0.069	−0.137 *
	Social identity	-	-	-	-	-	-	-	−0.035
	SDO	-	-	-	-	-	-	-	-

Index = index of evaluative difference between Norm adhering and Norm violating targets, Prescriptive norm, Descriptive norms, Fear of COVID-19, Disgust—Disgust, RWA = Right Wing Authoritarianism, SDO = social dominance orientation, Social identity. * *p* < 0.05, ** *p* < 0.01, *** *p* < 0.001, # *p* < 0.1.

**Table 7:** Unstandardized B’s, Betas, zero-order, and partial correlations between fear of COVID-19, disgust, RWA, SDO, social identity, prescriptive and descriptive norms, and index of target evaluations.

Model		Unstandardized Coefficients		Standardized Coefficients	*t*	Sig.	Correlations	
		B	Std. Error	Beta			Zero-Order	Partial
1	(Constant)	−73.210	85.005		−0.861	0.390		
	COVIDfear	−5.413	2.618	−0.151	2.067	0.040	0.052	−0.149
	Disgust	1.011	0.888	0.081	1.139	0.256	0.099	0.083
	RWA	0.777	0.459	−0.096	−0.861	0.093	0.037	0.122
	SDO	−1.457	0.978	−0.080	−1.100	0.272	−1.170	−0.085
	Social identity	0.664	0.665	0.070	1.384	0.319	0.103	0.073
	Prescriptive norms	7.973	3.05	0.331	4.097	0.000	0.255	0.286
	Descriptive norms	−3.1411	2.363	−0.101	1.329	0.185	0.055	−0.096
Dependent Variable: Index of Target Evaluations								

**Table 8:** Post-hoc regression of Unstandardized Bs, Betas, zero-order, and partial correlations between social identity, disgust, fear of COVID-19, SDO, RWA, personal norms, social norms, and index of target evaluations.

**Model**		**Unstandardized Coefficients**		**Standardized Coefficients**	***t***	**Sig.**	**Correlations**		
		**B**	**Std.** **Error**	**Beta**			**Zero-Order**	**Partial**	**Part**
1	(Constant)	−134.213	68.463		−1.960	0.051			
	Socid	0.680	0.583	0.070	1.166	0.245	0.128	0.074	0.068
	Disgust	1.450	0.733	0.133	1.978	0.049	0.154	0.125	0.115
	Cofear	2.466	2.297	0.070	1.074	0.284	0.129	0.068	0.062
	SDO	−0.676	0.954	−0.050	−0.709	0.479	−0.156	−0.045	−0.041
	RWA	−0.219	0.315	−0.049	−0.694	0.489	−0.055	−0.044	−0.040
	Personal Norms	20.070	3.670	0.347	5.469	<0.001	0.363	0.329	0.317
	Social Norms	−2.010	3.249	−0.038	−0.619	0.537	0.105	−0.039	−0.036
Dependent Variable: Index of Target Evaluations									

## Data Availability

The data is currently retained at the Univeristy of Otago (by J. A. Hunter—jackie.hunter@otago.ac.nz) and will be made available on request.
